# Supramolecular Approaches to Nanoscale Morphological Control in Organic Solar Cells

**DOI:** 10.3390/ijms160613381

**Published:** 2015-06-11

**Authors:** Alexander M. Haruk, Jeffrey M. Mativetsky

**Affiliations:** 1 Department of Physics, Applied Physics and Astronomy, Binghamton University, Binghamton, NY 13902, USA; 2 Department of Chemistry, Binghamton University, Binghamton, NY 13902, USA; E-Mail: aharuk1@binghamton.edu

**Keywords:** solar cells, organic semiconductors, morphology, supramolecular interactions, self-assembly, nanowires, device stability

## Abstract

Having recently surpassed 10% efficiency, solar cells based on organic molecules are poised to become a viable low-cost clean energy source with the added advantages of mechanical flexibility and light weight. The best-performing organic solar cells rely on a nanostructured active layer morphology consisting of a complex organization of electron donating and electron accepting molecules. Although much progress has been made in designing new donor and acceptor molecules, rational control over active layer morphology remains a central challenge. Long-term device stability is another important consideration that needs to be addressed. This review highlights supramolecular strategies for generating highly stable nanostructured organic photovoltaic active materials by design.

## 1. Introduction

Solar cells based on organic molecules offer a potentially low-cost, mechanically-flexible, and lightweight platform for the clean conversion of sunlight to electricity [1,2,3,4,5]. From a research and development standpoint, organic solar cells, or organic photovoltaics (OPV), offer several advantages that make them promising alternatives to their inorganic counterparts. The high extinction coefficient of organic semiconductors allows for a reduction in the amount of material needed to harvest incoming photons [1,6]. Chemical synthesis provides fine-tuning of the chemical structure, and in turn, the optical and electrical properties of the active materials [1,4,7,8]. These materials are also easily processed from solution near room temperature and have great potential for low-cost industrial scale manufacturing by roll-to-roll processing and spray-coating [2,9,10].

Over the past decade, the efficiency of lab-scale organic solar cells has more than doubled, to beyond 10% [11,12,13]. The highest performance organic solar cells rely on a bicomponent nanostructured active layer morphology. Despite the key role of this nanostructuring in dictating device performance [2,6,14,15,16,17,18,19,20,21], predictive control over active layer structure remains a pivotal challenge. Moreover, because nanostructuring is often achieved through the formation of metastable phases, active layer stability, especially at elevated temperatures, is also of concern [2,6,16,22].

Supramolecular, *i.e*., non-covalent, interactions offer a means of manipulating the assembly of organic systems [23,24,25,26,27,28]. Through judicious molecular design, specific molecular assembly motifs can be “programmed”. Supramolecular approaches have only recently begun to be applied to OPV systems, to promising effect. In this review, we examine supramolecular strategies for tailoring and stabilizing the nanoscale morphology of organic solar cell active layers. After introducing the basic operating principles of organic solar cells, we will highlight OPV systems that are designed to harness aromatic stacking, hydrogen bonding, and shape complementarity in order to improve the nanomorphology, performance, and stability of OPV active layers.

## 2. Working Principles of Organic Solar Cells

**Figure 1:**
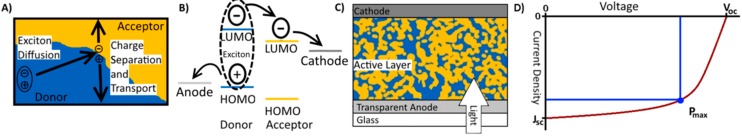
(**A**) Schematic of the main processes that take place in an organic solar cell; (**B**) Corresponding energy level diagram; (**C**) Bulk heterojunction solar cell structure; (**D**) Typical electrical characteristics of an organic solar cell with the *J*–*V* curve in red and the current density and voltage at *P*_max_ shown in blue.

Organic solar cell active layers consist of two organic semiconductors, one serving as an electron donor and the other as an electron acceptor. Absorption of a photon leads to the generation of an exciton, a short-lived bound electron-hole pair. Due to the high binding energy of excitons in organic semiconductors [2,16], the exciton cannot dissociate unless it diffuses to a donor–acceptor interface, where it is energetically favorable to separate into free charges (see Figure 1A,B) [2]. For the charges to be collected, the electron must travel through the acceptor to the cathode while the hole must travel through the donor to the anode [2,6,16,29,30].

For efficient organic solar cell operation, two competing length scale requirements must be satisfied: (1) the light absorbing layer must be hundreds of nanometers thick for efficient light absorption; and (2) the light-induced generation of excitons must occur within about 10 nm of a donor–acceptor interface for separation into free charge carriers [3,7,14,22], otherwise the light-generated excitation will be lost to recombination. The bulk-heterojunction concept [3,6,15,22,31] cleverly satisfies both requirements through the use of nanoscale interpenetrating donor and acceptor domains, as illustrated in Figure 1C. In bulk-heterojunction solar cells, the light-capturing film is sufficiently thick to absorb most incoming light, while the high density of donor–acceptor interfaces provides efficient charge generation. In practice, in addition to pure domains, finely mixed donor–acceptor regions are also commonly present on account of donor–acceptor miscibility [22,32,33,34,35]. Continuous charge transport pathways are required for holes to percolate through the donor to the anode, and electrons to percolate through the acceptor to the cathode. Molecular orientation, molecular packing structure, and the degree of intermolecular order also significantly impact charge transport efficiency [36,37,38,39,40,41,42,43,44,45]. Domain dimensions, continuity, and internal structure are therefore key features that govern OPV performance.

Solar cell performance is measured by recording current density *J*, *i.e*., current per device area, as a function of voltage *V* (see Figure 1D). The short-circuit current density *J*_sc_ is the current density when there is no voltage difference between the anode and cathode. The *J*_sc_ is influenced by the amount of light that is absorbed, the exciton separation efficiency, and the ability of the free charges to reach the electrodes [30]. The open circuit voltage *V*_oc_ is the voltage across the device when there is no net flow of current, and is predominantly dependent on the difference between the highest occupied molecular orbital (HOMO) of the donor and the lowest unoccupied molecular orbital (LUMO) of the acceptor, while also being influenced by the electrode work function [1,46,47,48,49]. The fill factor (FF), associated with the “squareness” of the *J*–*V* curve, is defined as the ratio of the maximum power density of the cell *P*_max_ and the theoretical maximum power density based on *J*_sc_ and *V*_oc_:

(1) $$FF=\frac{P_{\max}}{J_{\text{sc}}V_{\text{oc}}}$$

Power conversion efficiency (PCE) is the percentage associated with the ratio of the maximum power density and the power density of the incident light:

(2) $$PCE=\frac{P_{\max}}{P_{\text{in}}}\times 100\%$$

The series resistance *R*_s_ is the internal resistance of the device, and the shunt resistance *R*_sh_ is a measure of the resistance to current leakage in the device [7].

Nanostructured bulk heterojunction active layers are typically achieved by casting a thin film from a donor–acceptor co-solution, and using thermal annealing, additives, or other means to promote phase separation [2,22,50,51]. The resulting morphology is governed by a complex interplay between film formation kinetics, component miscibility, and crystallization, making the associated solar cell performance highly sensitive to the precise processing conditions [16,22,46,52]. The design of donor–acceptor systems with specified supramolecular interactions provides opportunities to tailor organic solar cell active layer structure, which plays such a critical role in device operation.

## 3. Aromatic Stacking

Aromatic stacking results from the interactions between π-orbitals of separate π-systems. For organic semiconductors, with their large, often planar, conjugated π-systems, this can result in a face–face interaction between the molecules. In this arrangement, charge transport is favored along the stacking direction [38,39,53,54,55,56]. This section will discuss how aromatic stacking has been harnessed to form nanowires, manipulate active layer domain size, and mediate donor–acceptor interactions.

### 3.1. Self-Assembled Nanowires

**Figure 2:**
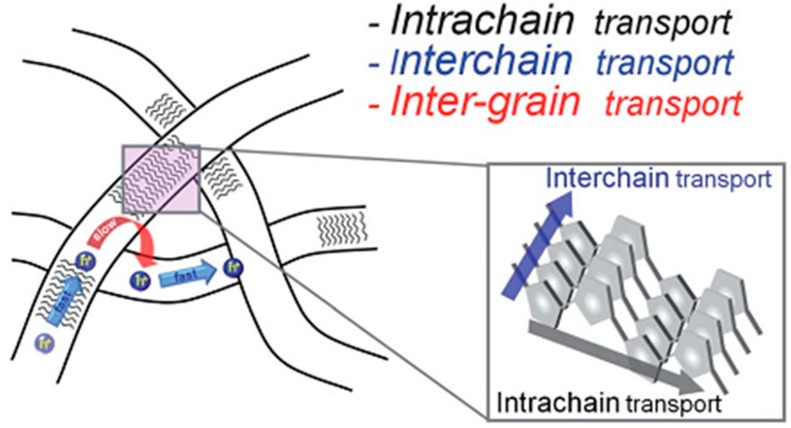
Schematic of the face-face stacking of polymer chains in P3AT nanowires. Reproduced from [58] with permission from The Royal Society of Chemistry.

Nanowires are promising for achieving high performance in organic solar cells as they present both a large surface-to-volume ratio, essential for charge separation, and a continuous conduit for charge collection. The most common methods for generating organic semiconductor nanowires involve the addition of a poor solvent, or gradual cooling of a warm saturated solution, in order to slowly induce aggregation and precipitation of the organic semiconductor [30,57,58,59,60,61,62,63,64]. A widely-studied class of polymer donor, poly(3-alkylthiophene) (P3AT), forms nanowires through face-to-face stacking of the polymer backbone, with the stacking direction being parallel to the long axis of the wire (see Figure 2) [30,58,65]. This long-range face-to-face packing leads to efficient hole transport along the wire axis [58,59,65].

**Figure 3:**
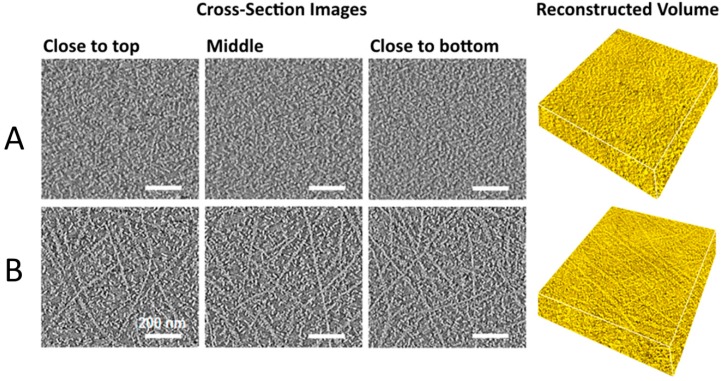
Cross-section images obtained by electron tomography from the top to bottom along the plane of the active layer for (**A**) a conventional bulk heterojunction and (**B**) a nanowire-based heterojunction, with reconstructed 900 × 900 × 200 nm volumes. Adapted from [60] with permission from the American Chemical Society. Scale bar: 200 nm.

As shown in Figure 3, in OPV active layers, P3AT nanowires form a network of continuous pathways for charge to travel through [30,57,58,59,60]. For a 100 nm thick active layer, poly-3-hexylthiophene (P3HT):phenyl-C_61_-butyric acid methyl ester (PCBM) devices that incorporated P3HT nanowires outperformed conventional bulk heterojunctions. The improved performance in the nanowire-based solar cells was proposed to stem from the 15-fold higher hole mobility in the active layer [60], which resulted in a near unity electron/hole mobility balance. A mismatch between electron and hole mobility can hinder device performance though a build-up of space charge [66,67].

As the active layer thickness was increased, conventional devices saw a worsening of the mobility imbalance and an increase in *R*_s_, while the nanowire devices maintained well-balanced electron and hole mobilities, and a low *R*_s_ [60]. In other words, the nanowire-based active layers better maintained percolation pathways for charge collection, even in relatively thick films. The *J*_sc_ of nanowire devices increased with thickness, up to 300 nm, while the *V*_oc_ and FF remained constant. The improved *J*_sc_ was attributed to the increased light absorption of the thicker active layer. The top-performing nanowire device had a PCE of 4.21%, 46% higher than the optimized conventional device [60]. This study demonstrates the potential merits of nanowire based solar cells: better charge percolation enables the use of thicker active layers, which in turn improves light absorption by the device. Active layers that are hundreds of nanometers thick are also more amenable to large-scale manufacturing than thinner layers [9,58].

Another important property of a nanowire is the aspect ratio, *i.e*., the ratio of length to diameter. Self-assembled nanowires formed from poly(3-butylthiophene)-*b*-poly(3-octylthiophene) diblock copolymers maintained a width of 13–16 nm, but had a composition dependent aspect ratio (ranging from 48 to 263), depending on the relative amount of each block. Active layers incorporating wires with the largest aspect ratio had the highest *J*_sc_, which was attributed to these wires providing superior charge transport and collection over longer length scales [59].

Nanowires have also been employed to improve the performance of lateral organic solar cells (LOSC), in which the cathode and anode are laterally spaced, with the active layer in between. This unconventional architecture has the advantage that it does not require a transparent electrode; however, efficiencies thus far have been limited, largely owing to the relatively long distances over which charges must be transported to reach the electrodes, typically hundreds of nanometers to tens of micrometers [68,69,70]. Due to the favorable charge transport properties and durability of P3HT nanowires, it was possible to make a flexible device with a PCE of 2% under low light intensity (0.1 Sun), that was able to retain 95% of its efficiency after extended stress testing [71]. Bimolecular recombination of charge carriers was a dominant mechanism for performance loss, likely owing to the long channel length over which electrons and holes must be transported for collection at the cathode and anode. This was particularly apparent under high light intensities, when there is a high density of charge carriers. It was hypothesized that the introduction of acceptor nanowires would help lower the amount of recombination under full illumination [71].

**Figure 4:**
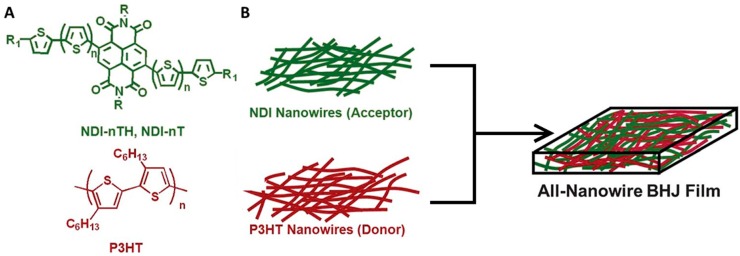
(**A**) Molecular structure of P3HT and NDI (*n* = 1–3, R_1_ = H or C_6_H_13_, R = ethylhexyl); (**B**) Schematic of an all-nanowire bulk heterojunction (BHJ) active layer. Adapted from [62] with permission from The Royal Society of Chemistry.

A primary challenge with making an all-nanowire active layers has been synthesizing acceptor nanowires of appropriate size, particularly for small molecule acceptors. A series of oligothiophene-functionalized naphthalene diimide (NDI) acceptors were synthesized with varying conjugation length and end groups. Nanowires formed from these compounds were combined with P3HT nanowires to create all-nanowire heterojunctions (Figure 4) [62].

The best performing NDI acceptor had tri-thiophene chains and hexyl end groups (NDI-3TH), providing the narrowest nanoribbons (80–250 nm wide, 14.2–15.3 nm thick, and 2–10 µm long), and the highest *V*_oc_, *J*_sc_, and FF. When combined with P3HT nanowires, the NDI-3TH wires produced devices with a PCE of 1.15%, 54% greater than optimized bulk-heterojunctions comprising the same molecules [62,72]. This significant increase was attributed to the nanoribbons having narrow domains, which aid in exciton separation, and a bicontinuous network morphology, for effective charge percolation of both electrons and holes.

### 3.2. Tuning Bulk Heterojunction Domain Size

The creation of appropriately sized domains is of vital importance to OPV performance. If the domains are too large, a large fraction of light-generated excitons will recombine before reaching a donor–acceptor interface where the exciton can be split into free charges. If the domains are too small, then the free charges are less likely to reach the electrodes due to charge trapping.

**Figure 5:**
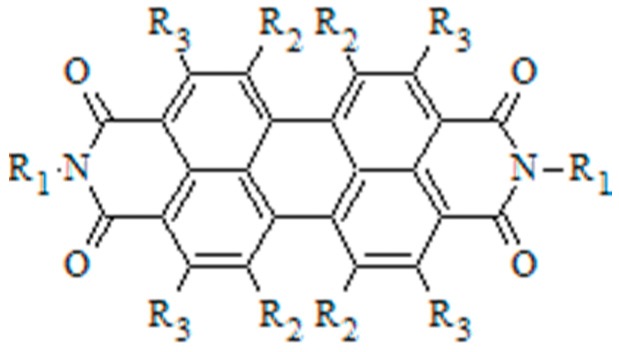
Perylene diimide (PDI) structure with imide (R_1_), bay (R_2_), and headland (R_3_) positions labeled.

Although fullerenes have historically outperformed other acceptor molecules, there is significant interest in exploring alternatives [73,74,75,76,77,78]. Perylene-based dyes have attracted attention due to their simple synthesis, their low-lying LUMO levels, their light absorption in the visible range, their ability to self-assemble, and their favorable electron transport properties [63,74,79]. Planar perylene diimide (PDI) molecules (Figure 5) have a strong propensity to self-assemble by π–π stacking to form highly crystalline fibers featuring superior electron mobilities [63,79,80,81,82]. The dimensions of PDI fibers can be tuned somewhat by altering the processing conditions. However, generally, PDI forms fibers with widths ranging from hundreds of nanometers to several micrometers [63,80,83,84,85,86,87,88,89,90,91,92]. When used as an acceptor in bulk heterojunction active layers, PDIs domains are typically too large for efficient exciton separation [93,94,95,96], resulting in limited efficiencies [97,98].

**Figure 6:**
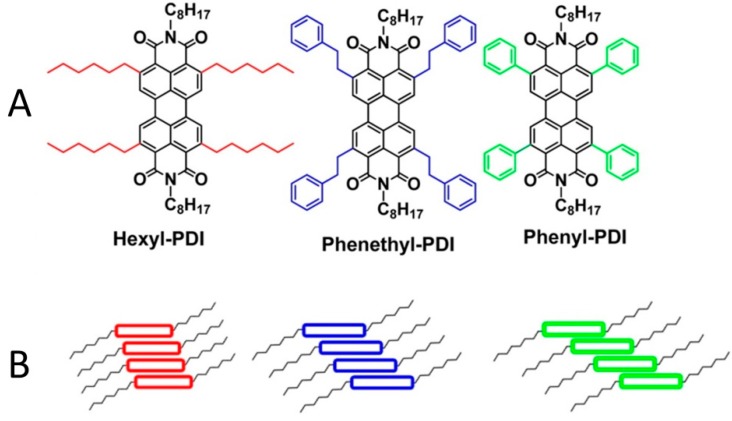
(**A**) Molecular structure of PDI derivatives and (**B**) the hypothesized degree of slip-stacking. Adapted from [99] with permission from the American Chemical Society.

One strategy for tuning the assembly of PDI molecules is to alter the amount of lateral offset between the stacked PDI molecules, by adding functional groups such as hexyl, phenethyl, or phenyl groups to the four headland positions (see Figure 6) [99].

In pure films, the hexyl-PDI produced large (1–2 μm) crystalline fibers and the roughest film structure. The phenethyl-PDI formed the smoothest pure film and was the least crystalline. The phenyl-PDI was between the hexyl and phenethyl in terms of crystallinity and roughness of the pure film [99]. A similar trend was observed when these acceptor molecules were blended with polymer donor poly(bithiopheneimide terthiophene) (PBTI3T), though the blend films were smoother and exhibited a reduced crystallinity relative to the pure PDI films. It was the phenyl-PDI-based active layer, and its 2–5 nm crystalline grains, that had the highest *J*_sc_ and an average PCE of 3.60% [99].

**Figure 7:**
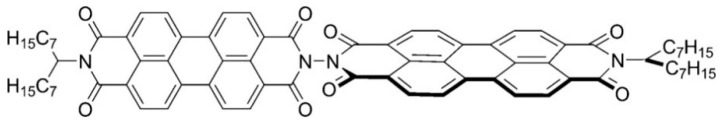
Twisted PDI dimer. Adapted from [100] with permission from the American Chemical Society.

Another approach to mediating the assembly of PDI and limiting PDI domain size in OPV active layers is to form dimers. PDI dimers linked through the imide position have been synthesized, as shown in Figure 7 [100,101]. The PDI moities are rotated with respect to each other due to the steric repulsion of the imide carbonyls. In blended films with polymer donor poly[4,8-bis-(2-ethylhexyloxy)-benzo[1,2-b:4,5-b′]dithiophene-2,6-diyl-alt-4-(2-ethylhexyloxy-1-one)thieno[3,4-b]thiophene-2-yl-2-ethylhexan-1-one] (PDBTTT-CT), PDI monomer domains were several micrometers in size while PDI dimer domains were on the order of 10 nm. A sizeable concurrent increase in OPV performance was obtained, with the *J*_sc_ increasing from 0.8 to 9.0 mA/cm^2^ and the PCE increasing from 0.13% to 3.20% [100,101].

PDI dimers have also been made by attachment at the bay positions. A single covalent link between the monomers allowed for rotation about the bond, which disrupted aggregation. Increasing the number of covalent links between the monomers created more rigid and planar systems that readily aggregate through π-stacking. The reduction in domain size for the singly-linked PDI dimers led to an enhancement of the *J*_sc_ and a maximum PCE of 3.63% [102].

Very recent studies have used bay-linked PDI dimers to limit the PDI domain size and achieve efficiencies in excess of 6.0% [103,104]. In the best-performing system, a PDI dimer bearing a bulky spiro-fluorene linking group produced domain sizes of 20–30 nm along with a large *V*_oc_ of 0.98 V when combined with a difluorobenzothiadiazole-quarterthiophene polymer donor, PffBT4T-2DT. It has also been shown that the use of a conjugated fused ring linkage can have similar results due to the combination of domain size reduction and energy level shift [105]. The tunable assembly and concomitant improvements in photovoltaic performance demonstrated with PDI derivatives show great promise for π-stacking acceptor molecules.

### 3.3. Selective Donor–Acceptor Interactions

**Figure 8:**
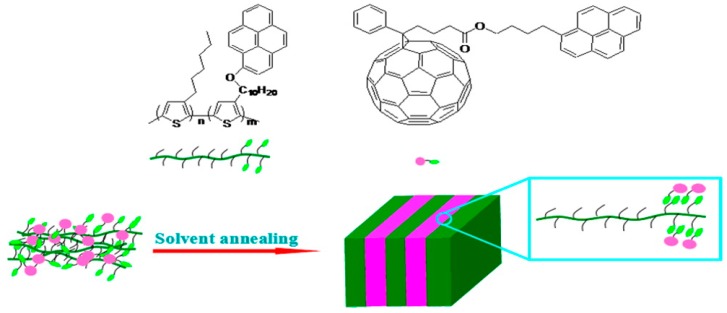
Chemical structures of copolymer donor P3HT-*b*-P3TPy and fullerene acceptor PCPy, and schematic of the self-assembled lamellar active layer structure. Reproduced from [106] with permission from the American Chemical Society.

Aromatic stacking has also been employed to mediate interactions between donors and acceptors in OPV active layers. As shown in Figure 8, pyrene (Py) groups were attached to a polythiophene to create block copolymers with different ratios of hexyl- and Py-containing blocks [106]. When combined with a Py-containing fullerene derivative (PCBPy) and formed into films using a self-assembly-promoting solvent annealing process, the Py groups of the copolymer and fullerene were able to π-stack, as evidenced by the presence of a new X-ray diffraction peak for the copolymer-fullerene film comprising the 3:1 hexyl:Py copolymer. Transmission electron microscope (TEM) and atomic force microscope (AFM) characterization revealed that as the amount of Py was increased, the morphology changed from one resembling a typical P3HT:PCBM film to a lamellar structure, with 10–20 nm wide domains of alternating PCBPy rich and poor regions [106].

### 3.4. Covalently Attached Donors and Acceptors

Another strategy for manipulating the assembly of donor–acceptor systems is to covalently attach donor and acceptor small molecules [107,108,109], oligomers [110,111,112], or polymers [113,114,115,116,117,118]. In some instances, aromatic stacking has led to fibrous coaxial donor–acceptor assemblies [108,115]. When incorporated into organic solar cells, donor–acceptor dyads or copolymers have primarily been used as a third component that can increase the stability of the active layer [114,116], limit macrophase separation, and increase performance [111,113,114,116]. So far, when used as the sole component of an active layer, most covalently attached donor–acceptor systems have exhibited limited OPV performances [109,112,114,117,118].

Recent work with donor–acceptor block copolymers has shown significant promise. When incompatible polymer blocks are covalently linked to form a block copolymer, the blocks phase separate, giving rise to a rich composition-dependent phase behavior that can produce films with lamellar, sphere, cylinder, and gyroid morphologies and tunable nanoscale characteristic lengths [119,120,121]. Although such nanoscale structuring is highly desirable for organic solar cells, the use of rigid, fully conjugated donor–acceptor block copolymers—for which competition between segregation and crystallization can complicate the self-assembly behavior—are still relatively unexplored [120,122,123,124].

A fully conjugated block copolymer made from P3HT and a polymer acceptor poly-((9,9-dioctylfluorene)-2,7-diyl-alt-[4,7-bis(thiophen-5-yl)-2,1,3-benzothiadiazole]-2′,2″-diyl) (PFTBT) was shown to be close to three times more efficient than a bulk-heterojunction made from the homopolymers; the PCE for the copolymer was 2.7%, compared to 1.0% PCE for the homopolymer blend [125]. The improved performance in the copolymer system was attributed to the lamellar structure of the active layer, the face-on orientation of the copolymer, and the conjugation across the donor–acceptor interface.

**Figure 9:**
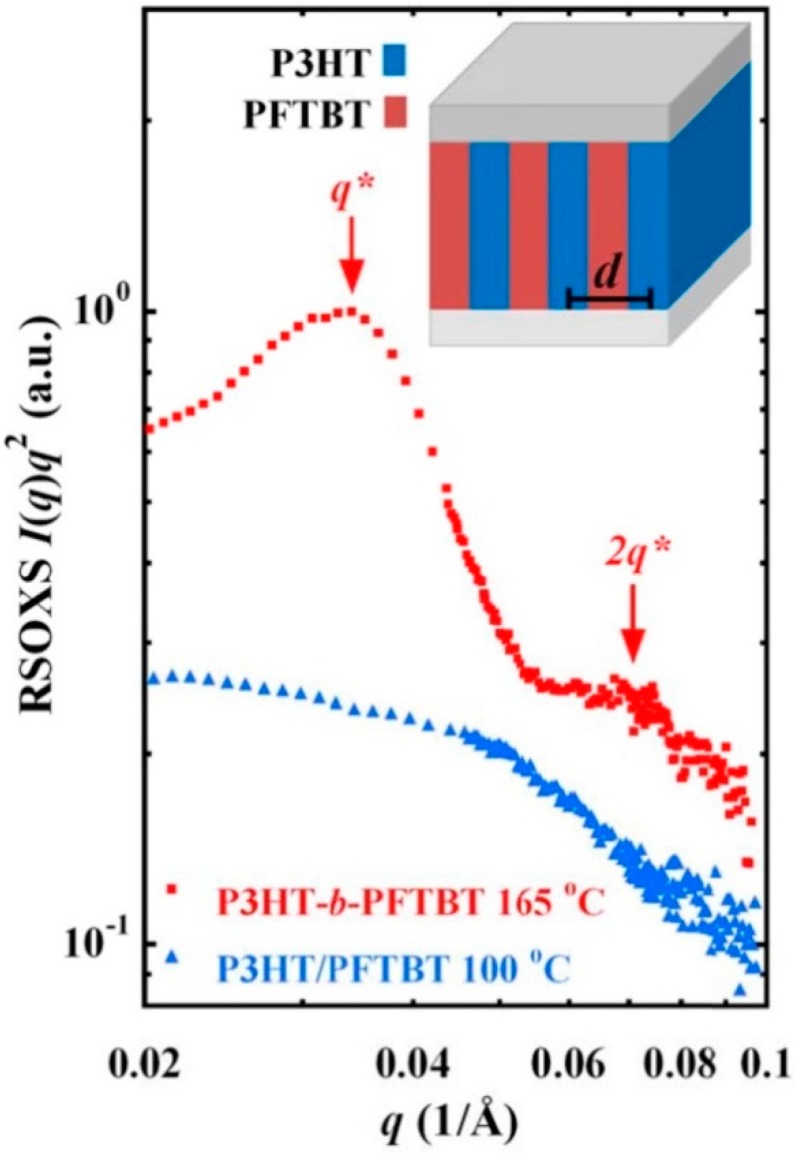
Resonant soft X-ray scattering data showing a well-defined primary scattering peak, q*, and a weak second-order peak, 2q*, indicating the presence of lamellar domains in donor–acceptor block copolymer P3HT-*b*-PFTBT, with a period of *d* = 18 nm. Reproduced from [125] with permission from The American Chemical Society.

Resonant soft X-ray scattering was employed to reveal the lamellar morphology of the active layer, featuring alternating donor and acceptor lamella, oriented normal to the substrate (Figure 9). The width of the individual lamella, about 9 nm, corresponds well with the expected exciton diffusion length, while the vertical orientation of the lamella is well-suited for charge collection. In contrast with the typical edge-on orientation of P3HT in organic solar cell active layers [126,127,128,129], the P3HT block of the copolymer exhibited a preferential face-on orientation, *i.e*., with the molecular plane nearly parallel to the substrate. It was hypothesized that the preferred orientation of the P3HT resulted from the interaction of the PFTBT with the underlying poly(3,4-ethylenedioxythiophene):polystyrene sulfonate (PEDOT:PSS) hole transport layer [130] which led to a face-on orientation for the PFTBT; since the P3HT was linked to the PFTBT, it adopted a similar orientation [125]. A face-on molecular orientation provides efficient out-of-plane charge transport, along the molecular stacking direction, to the charge collecting electrodes [38,39,53,54,55,56]. Finally, the nature of the coupling between donor and acceptor components significantly impacts the exciton dissociation process [131]; in this instance, the conjugated link between the donor and acceptor appears to be more effective at generating charge than the intermolecular interface between donor and acceptor homopolymers.

## 4. Hydrogen Bonding

Hydrogen bonding, being a directional and relatively strong supramolecular interaction between hydrogen and polar groups, is promising for mitigating self-assembly. Nature, of course, makes extensive use of hydrogen bonds for generating secondary protein structures, such as alpha helices or beta sheets, and the helical structure of DNA. In this section we discuss the use of hydrogen bonding to mediate self-assembly and stabilize OPV active layers.

### 4.1. Tuning Self-Assembly with Hydrogen Bonding

**Figure 10:**
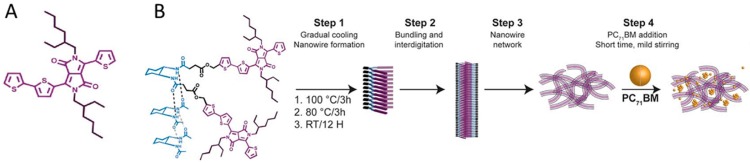
(**A**) Structure of diketopyrrolopyrrole (DPP); (**B**) Scheme for generating hydrogen-bonded diketopyrrolopyrrole (DPPHP) nanowires. Adapted from [132] with permission from The Royal Society of Chemistry.

In some OPV systems, the donor and acceptor become finely mixed and do not form networks of nanoscale domains that are needed for efficient charge photogeneration and collection. For the thiophene-capped diketopyrrolopyrrole (DPP) small molecule donor shown in Figure 10A, pure DPP films prepared using a stepwise procedure for promoting self-assembly exhibited crystalline grains; however, upon introduction of a fullerene acceptor, phenyl-C_71_-butyric acid methyl ester (PC_71_BM), DPP assembly was disrupted, resulting in an OPV efficiency of 0.24% [132]. On the other hand, a hairpin-shaped diketopyrrolopyrrole (DPPHP), formed through the covalent attachment of *trans*-1,2-diamidocyclohexane onto DPP, was able to synergistically hydrogen bond and cofacially π-stack to form nanowires (see Figure 10B) [132]. Upon addition of PC_71_BM, the DPP nanowires remained intact, as confirmed by AFM and TEM. Solar cells comprising DPPHP nanowires and PC_71_BM exhibited an efficiency of 0.53%, more than double that of DPP devices that did not benefit from hydrogen bonding, and 400 times greater than devices in which DPPHP was not induced to self-assemble prior to fullerene addition [132].

Another study highlights the potential of employing competing π–π and hydrogen bonding interactions for optimizing active layer morphology [133]. Both hydrogen bonding and non-hydrogen bonding DPP compounds, with amide (a-DPP) and ester (e-DPP) side chains, respectively, were synthesized. When blended with PC_71_BM in films, the e-DPP produced crystalline π-stacked aggregates, about 200 nm in size, while the hydrogen bonding a-DPP maintained a much smaller domain size and a reduced degree of crystallinity. Despite the small domain size and limited molecular order, active layers incorporating the a-DPP had close to twice the hole mobility compared with the e-DPP. This favorable combination of small domain size and efficient charge transport in the a-DPP-based active layer resulted in a dramatically higher *J*_sc_, 11.0 mA/cm^2^, compared to 3.9 mA/cm^2^ in the e-DPP, and a PCE of 3.65%. After optimization through the addition of nitrobenzene, the performance of devices comprising a-DPP was further increased to 4.57% PCE owing to an increase in the *J*_sc_ to 12.6 mA/cm^2^ [133].

**Figure 11:**
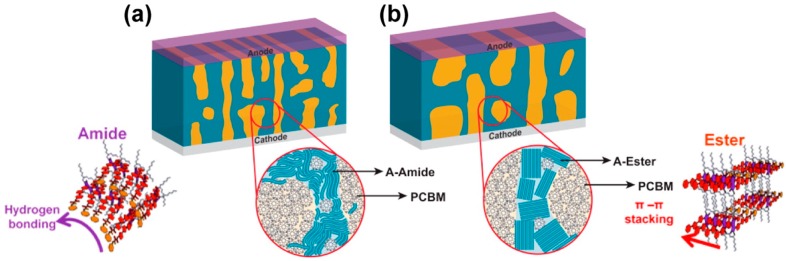
Packing structure and active layer morphology for: (**a**) amide-DPP; and (**b**) ester-DPP. Adapted from [133] with permission the American Chemical Society.

The improved performance of the a-DPP was attributed to how the molecule assembled to form short fiber-like interconnected domains, through hydrogen bonding (see Figure 11), that could effectively transport charge. These results support the notion that domain continuity is of paramount important to device performance [134]. The ubiquity of π-interactions in OPV systems opens up the possibility of using competing hydrogen bonding interactions as a general route for disrupting unwanted domain coarsening [133]. Further work is needed, however, to better predict the conditions under which these intermolecular interactions are competitive or synergistic.

### 4.2. Active Layer Stabilization

For organic solar cells to become a viable technology, the active layer’s structure must remain stable under a range of operating conditions. Methods for limiting structural evolution of the active layer are therefore of great importance [50,135,136,137].

Hydrogen bonding between donor and acceptor has been used to control the distribution and stability of fullerene aggregates in solar cell active layers. An electron donating block copolymer of P3HT-*b*-poly[3-(2,5,8,11-tetraoxadodecane)thiophene] (P3HT-*b*-P3TODT) was synthesized and blended with electron acceptor bisphenyl C_61_-butyric acid (bis-PCBA), allowing hydrogen bonding to take place between the tetraether sidechain of the P3TODT block and the carboxylic acid of the bis-PCBA [138]. The copolymer formed alternating P3HT and P3TODT lamella in blends comprising up to 40 wt % bis-PCBA. TEM experiments indicated that the bis-PCBA acceptor selectively segregated to the P3TODT regions where hydrogen bonding between the donor and acceptor can take place. This result was supported by the infrared spectra of the blend, which show a shift and a splitting of the carboxylic acid peak, suggesting the presence of hydrogen bonding through a carboxylic acid–ether interaction.

The performance of the P3HT-*b*-P3TODT:bis-PCBA device with hydrogen bonding was twice that of a non-hydrogen bonding P3HT-*b*-P3TODT:PCBM device, but was lower than the P3HT:PCBM reference system, with PCEs of 2.04%, 0.95%, and 3.01%, respectively. Accelerated aging tests, however, showed significant improvements in the stability of the hydrogen bonded system: the P3HT-*b*-P3TODT:bis-PCBA retained 46% of its PCE after being subjected to an elevated temperature of 150 °C for six hours, while the P3HT:PCBM reference device only retained 13% of its PCE under the same conditions. This enhanced stability was attributed to a reduced diffusion of the fullerene due to the fullerene being hydrogen bonded to the copolymer donor [138].

**Figure 12:**
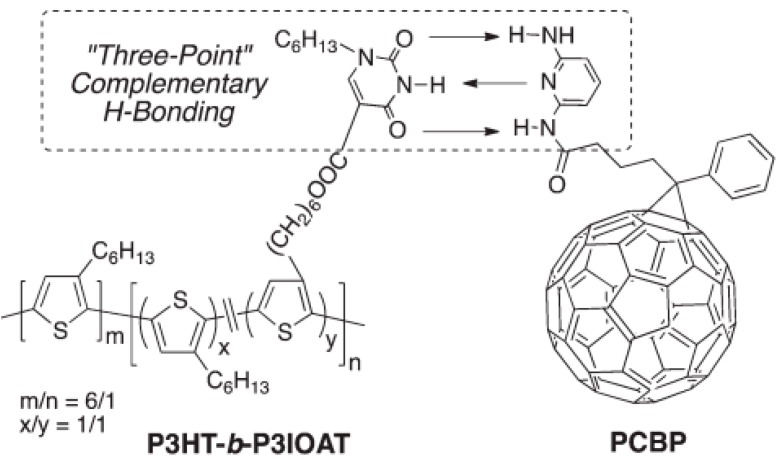
Structure of copolymer donor P3HT-*b*-P3IOA and fullerene acceptor PCBP, along with the three-point hydrogen bonding between the compounds. Reproduced from [141] with permission from The Royal Society of Chemistry.

In another study looking to increase stability, a “three-point” complementary hydrogen bonding scheme was employed, in which three hydrogen bonds are simultaneously formed between donor and acceptor [139,140,141]. Using a polythiophene block copolymer functionalized with isoorotic acid (P3HT-*b*-P3IOA) and diaminopyridine tethered fullerene derivative (PCBP) shown in Figure 12 [141], it was found that bulk heterojunction devices with the hydrogen-bonded derivatives were highly resistant to structural change, as evidenced by accelerated aging tests, done at 110 °C for 112 h [139]. The non-hydrogen bonding P3HT:PCBM reference device retained less than 40% of its PCE, while devices that incorporated components that were able to form one (P3HT-*b*-P3IOA:PCBM) or three (P3HT-*b*-P3IOA:PCBP) hydrogen bonds retained 65% and 75% of their initial PCE, respectively.

In a follow-up study, P3HT-*b***-**P3IOA nanowires were formed through solvent-induced precipitation [140]. The addition of fullerene acceptor PCBP led to an increase in the nanowire diameter, indicating that the PCBP was hydrogen bonding to P3HT-*b***-**P3IOA at the surface of the nanowire to generate a core-shell structure. When compared with active layers comprised of P3HT nanowires and PCBM, the copolymer system exhibited a significant reduction in the proportion of edge-on-oriented molecules, *i.e*., fewer molecules were oriented with the molecular plane nearly perpendicular to the substrate. This difference in molecular orientation between the two systems was attributed to the difference in the side-chain interaction with the PEDOT:PSS substrate [141]. A reduction in the proportion of edge-on oriented molecules is expected to facilitate out-of-plane charge transport to the anode and cathode [38,55,56,126,127,142,143,144,145,146]. When subjected to accelerated aging, the hydrogen bonded nanowire system was more stable, retaining 80% of the PCE after 112 h at 110 °C while the reference nanowire P3HT:PCBM device retained only 60% of its PCE after the same treatment [140].

### 4.3. Molecular Additives

**Figure 13:**
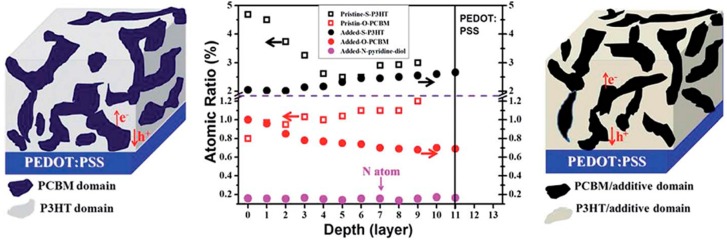
XPS Depth profile analysis of unannealed P3HT:PCBM films with and without additive. Reproduced from [152] with permission from The Royal Society of Chemistry.

Certain molecular additives have been shown to effectively promote the formation of nanostructured active layers, while simplifying device fabrication, e.g., circumventing the need for annealing [116,147,148,149,150,151]. 2,3-Pyridinediol is an interesting case, as it can hydrogen bond with the ester of PCBM while also π-stacking with the P3HT backbone [152]. This additive was found to increase the photovoltaic performance of P3HT:PCBM solar cells and allow for the use of relatively thick active layer films (350 nm). These improvements were associated with the presence of bicontinuous donor and acceptor nanodomains, about 30 nm in width, and an altered distribution of P3HT and PCBM throughout the active layer. Interfacial segregation during active layer formation commonly leads to a gradient in the proportion of donor and acceptor at different film depths [153,154,155]. In bulk-heterojunction P3HT:PCBM active layers, the top surface can consist of P3HT concentrations as high as 97 wt % [153,154,155,156]. As shown in Figure 13, addition of 2,3-pyridinediol led to a more evenly distributed vertical composition measured by X-ray photoelectron spectroscopy (XPS), with the sulfur content being representative of the P3HT, the oxygen of the PCBM, and the nitrogen of the 2,3-pyridinediol itself [152]. A more uniform distribution of donor and acceptor throughout the depth of the film is expected to lead to improved charge percolation.

The same additive was also effective for boosting the efficiency of P3HT:indene-C_60_ bisaduct and PTB7:PC_71_BM OPV devices with their respective PCE increasing from 3.35% to 5.93% and 5.46% to 7.54%, without and with the additive [152]. Furthermore, 2,3-pyridinediol led to improved device stability relative to the more commonly used additive 1,8-diiodooctane (DIO) in the PTB7:PC_71_BM device, with only a 3% drop in PCE, from 7.54% to 7.30%, after heating at 100 °C for 30 minutes, relative to a 36% drop in PCE, from 6.62% to 4.26%, for DIO.

## 5. Shape Complementarity

Shape complementarity is the principle of combining geometrically complementary molecules. This approach can be used to generate interlocking assemblies with intimate contact between donor and acceptor.

**Figure 14:**
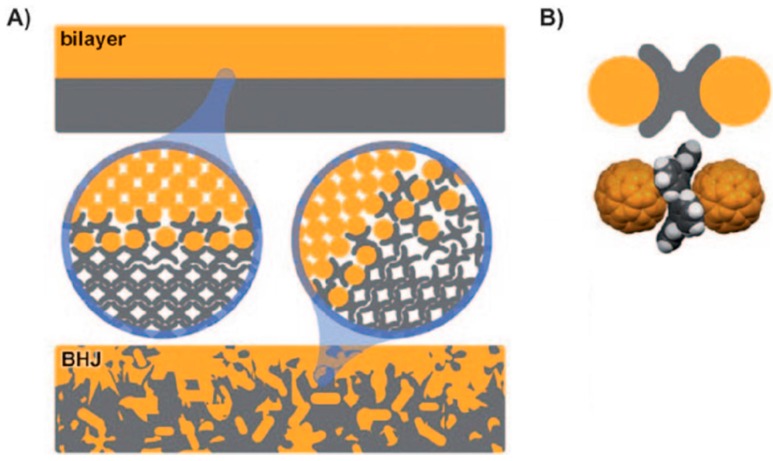
(**A**) Depiction of ball-and-socket interfaces in bilayer and bulk-heterojunction devices; (**B**) schematic depiction and molecular structure of the interface between c-HBC and C_60_. Adapted from [157] with permission from John Wiley and Sons.

**Figure 15:**
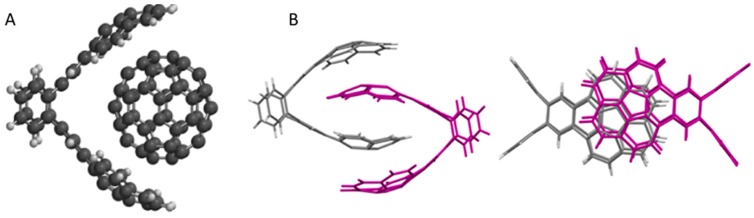
(**A**) Concave-concave buckycatcher around C_60_; (**B**) Interdigitated concave-concave buckycatchers. Adapted from [161] with permission from the American Chemical Society.

Contorted hexabenzocoronenes (c-HBC) have been combined with fullerenes, to produce ball-and-socket assemblies, as depicted in Figure 14 [157]. XPS measurements show the carbon 1s peak shifting to higher energy, and narrowing, which is consistent with improved charge transfer at the donor–acceptor interface. This improved charge transfer drives an enhancement in performance: c-HBC:C_60_ devices exhibit an efficiency that is 14 times greater than devices comprised of flat HBC analogues [157], while devices comprising contorted dibenzotetrathienocoronenes (DBTTC) and C_60_ have produced a PCE as high as 2.7% [158]. This efficiency is remarkable considering that neither the c-HBC nor the C_60_ absorb light appreciably in the visible wavelength range. This type of transparent solar cell has exciting potential for integration into light-harvesting windows. Other types of shape matching systems with potential applications for OPVs are also gaining attention, such as the buckycatchers shown in Figure 15 [159,160,161].

## 6. Conclusions

Supramolecular interactions are an effective tool for tuning the nanoscale morphology of OPV active layers and improving OPV performance. Nanowires formed through aromatic stacking exhibit narrow domains that benefit charge photogeneration and provide continuous pathways for charge collection, while allowing for the use of thicker active layers that absorb more light. Limiting the self-assembly of π-stacking small molecules through the addition of side chains, or the formation of dimers, has been effective for tuning domain size, resulting in some of the highest documented efficiencies for non-fullerene acceptors. Block copolymers have also been used to assemble appropriately-sized donor and acceptor domains with an alternating lamellar arrangement. Hydrogen bonding was shown to be effective for mediating interactions between OPV components, while simultaneously making the active layer more resistant to structural change during accelerated aging tests. Moreover, shape complementarity enabled performance improvements through intimate interfacial contact between donor and acceptor. Taken together, these results show the tremendous promise of supramolecular approaches for tailoring organic solar cell active structure with unprecedented control and predictability.
